# From Visible to Near–Infrared Light–Triggered Photochromism: Negative Photochromism

**DOI:** 10.3390/molecules29010155

**Published:** 2023-12-26

**Authors:** Ruiji Li, Bingzhao Mou, Mihoko Yamada, Wei Li, Takuya Nakashima, Tsuyoshi Kawai

**Affiliations:** 1 School of Pharmacy, Jining Medical University, Rizhao 276826, China; mbzd2001@163.com (B.M.); lwlw1919@163.com (W.L.); 2 Division of Materials Science, Nara Institute of Science and Technology (NAIST), Ikoma 630-0192, Japan; 3 Department of Chemistry, Graduate School of Science, Osaka Metropolitan University, Sumiyoshi, Osaka 558-8585, Japan

**Keywords:** photochromism, negative photochromism, visible to near–infrared light response, design strategy

## Abstract

Photochromic compounds, whose key molecular properties can be effectively modulated by light irradiation, have attracted significant attention for their potential applications in various research fields. The restriction of photoisomerization coloration induced by ultraviolet light limits their applications in the biomedical field and some other fields. Negative photochromism, wherein a relatively stable colored isomer transforms to a colorless metastable isomer under low–energy light irradiation, offers advantages in applications within materials science and life science. This review provides a summary of negatively photochromic compounds based on different molecular skeletons. Their corresponding design strategies and photochromic properties are presented to provide practical guidelines for future investigations. Negatively photochromic compounds can effectively expand the range of photochromic switches for future applications, offering unique properties such as responsiveness to visible to near–infrared light.

## 1. Introduction

**Scheme 1:**
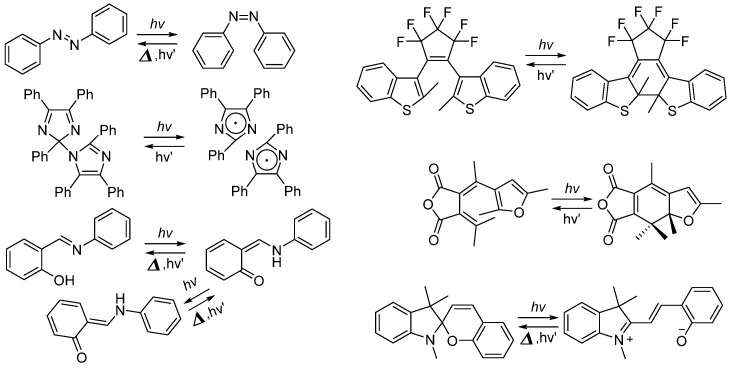
Photochromic reactions of typical organic photochromic compounds.

Photochromism refers to the reversible transformation of a molecular entity between two forms, A and B, having different absorption spectra, induced in one or both directions by light irradiation. The spectral change produced is typically, but not necessarily, of visible color and is accompanied by differences in other physical properties [1]. Until now, chemists have developed various kinds of photochromic compounds. However, the primary focus of recent research on organic photochromic compounds encompasses diarylethene (DAE) [2], spiropyran [3], azobenzene [4], hexaarylbiimidazole (HABI) [5], fulgide [6], Donor–Acceptor Stenhouse Adducts (DASAs) [7], and others (Scheme 1). According to their typical properties, photochromic compounds can be categorized based on the type of photochemical reaction and the thermal stability of both isomers, before and after light irradiation.

According to the type of photochemical reaction, photochromic molecules are classified into several categories: *trans*–*cis* photoisomerization processes, exemplified by azobenzene derivatives; homolytic cleavage processes, like HABI and PIC derivatives; proton transfer processes, seen in derivatives of salicylideneaniline and dinitrobenzylpyridine (DNBP); cyclization–cycloreversion reaction processes, represented by spiropyran, fulgide, and DAE.

Meanwhile, based on the thermal stability of the isomers generated by light irradiation, photochromic compounds can also be divided into T– and P–types [8]. If the photogenerated isomers spontaneously revert to their initial forms in the dark, they are classified as T–type photochromic molecules. Examples include azobenzene, spiropyran, HABI, and DASA. On the other hand, if the photogenerated isomers are thermally stable and revert to the initial isomers solely through light irradiation, are called P–type photochromic molecules, including DAE and fulgide. The most photochromic compounds undergo photochromic reactions, transitioning from thermodynamically stable colorless isomers to metastable colored isomers upon light irradiation. These compounds are referred to as positive–type photochromic compounds. Furthermore, photochromic molecules that undergo isomerization reactions upon light excitation, from stable colored isomers to their corresponding metastable colorless isomers, are recognized as negative photochromic molecules. Negative photochromism promotes color–fading reaction upon exposure to visible or near–infrared light irradiation. The backward reaction occurs with short–wavelength light irradiation or thermal coloration in the dark. The term negative photochromism has also been extended to systems wherein the intensity of the long wavelength absorption band of a dye has decreased upon light irradiation, with the emergence of a new band at a shorter wavelength in the UV light region [9,10]. Even though negatively photochromic compounds are not as well–known as their positive counterparts, their ability to undergo photoisomerization reactions with low–energy light irradiation holds promising application in the fields of biomedicine and materials science. This review paper provides an introduction and practical guidelines concerning negatively photochromic compounds. Emphasizing molecular structure, molecular design strategy, and color change, it offers fundamental insights into photochemical properties and elucidates the potential future applications.

## 2. Negatively Photochromic Compounds

### 2.1. Diarylethene

The DAE compounds involve *cis*–stilbene structure in their scaffold. They undergo a conrotatory peri–cyclization based on the Woodward–Hoffmann rules under UV light irradiation. Generally, the initial colorless open isomer of a DAE compound transforms to a colored closed isomer upon UV light irradiation [2]. That is, DAE compounds exhibit typical positive photochromism with significantly high fatigue resistivity and typical P–type photochromic nature.

**Scheme 2:**
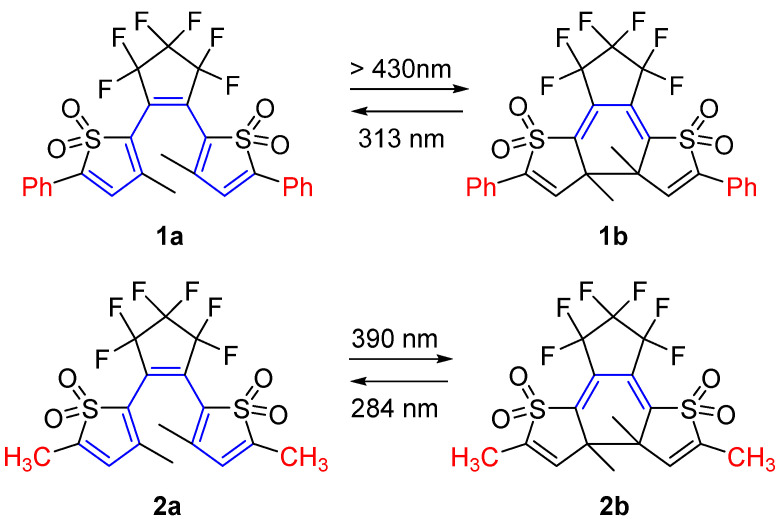
Molecular structures and photochromism of DAEs **1** and **2** (Blue color indicates the π–conjugation length; Red color indicates different substituents).

In 2008, Irie and coworkers reported a negatively photochromic compound based on the DAE skeleton [11]. The molecular design strategy involved connecting oxidized thiophene rings to the central ethene moiety through the 2–position (Scheme 2). The open isomer, which has a relatively longer π–conjugation length, displays an absorption maximum for **1a** at 356 nm in a faintly yellow 1,4–dioxane solution. Upon visible light irradiation (λ > 430 nm), a new absorption band emerges in the UV region, accompanied by a decoloration process. The photoinduced cyclization reaction quantum yield of **1a** in 1.4–dioxane is 1.9 × 10^−4^. Moreover, when the 1,4–dioxane solution of **1b** is irradiated with UV light (λ = 313 nm), it transforms from colorless to pale yellow. This shift coincides with the appearance of the absorption band at 356 nm, corresponding to the open isomer **1a**. Such behavior is emblematic of negative photochromism.

By substituting the phenyl units with methyl groups at the 5–position of both oxidized thiophene units, DAE compound **2a** in 1,4–dioxane exhibits no noticeable color change, a phenomenon termed “invisible photochromism”. The photoinduced cyclization reaction quantum yield of **1a** in 1.4–dioxane is 0.01. The unconventional photochromic behaviors of compounds **1** and **2** have also been elucidated through numerical calculation based on the density functional theory (DFT) [12].

**Scheme 3:**
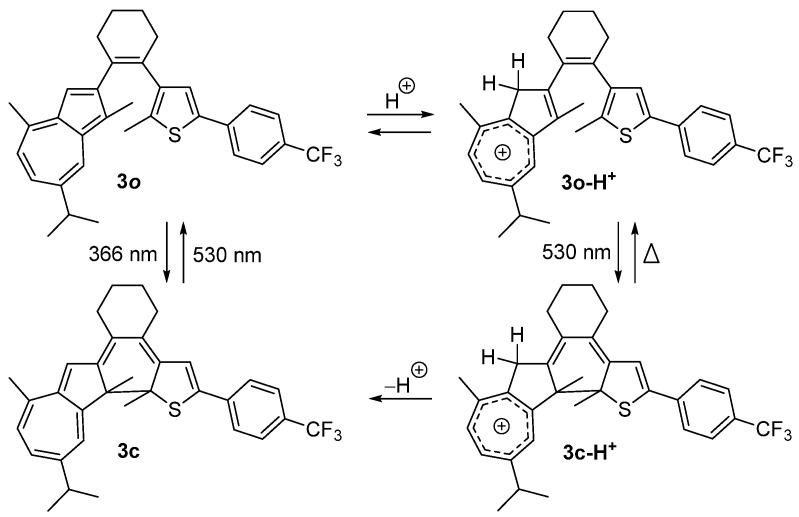
Photochromism of compound **3** and its acid–treated derivative.

Hecht and coworkers have also reported a negative photochromic compound based on the DAE skeleton, compound **3** [13]. Compound **3** exhibits a typical positive photochromic reaction in solution. However, after treatment with TFA, the absorption band of **3o** decreased to around 300 nm, and a new strong absorption band appeared, spanning from 350 to 550 nm. When irradiated with visible light at a wavelength of 530 nm, a color–fading reaction was observed in the **3o-H^+^** in cyclohexane solution. The absorption band in the visible region decreased, and another absorption band in the ultraviolet region became highly prominent. This behavior indicates the negative photochromism of the protonated compound, **3o-H^+^** (Scheme 3). Protonation of the azulene moiety largely improves the photosensitivity. This research paves the way for designing negative DAE compounds activated by visible light excitation.

**Scheme 4:**
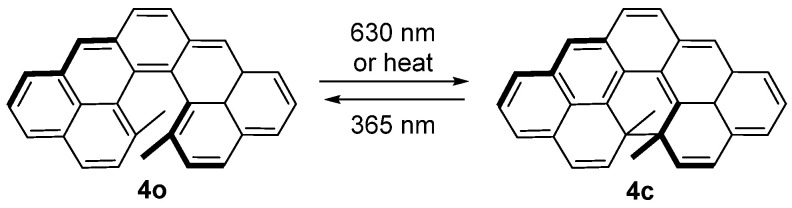
Photochromism of compound **4**.

Cethrene, a C–shaped hydrocarbon composed of seven fused benzenoid rings, might also be recognized as a DAE derivative. The blue solution of the metastable open isomer (**4o**) transformed into a colorless solution of the stable closed isomer upon irradiation at 630 nm (Scheme 4). The reversible photoreaction from colorless closed isomer to colored open isomer can be induced with 365 nm light irradiation [14].

### 2.2. Azobenzene (or Stilbene) and Derivatives

*Trans*–*cis* (or *E*–*Z*) isomerization is the distinguishing feature for the compounds of carbon–carbon or nitrogen–nitrogen double bonds. The most typical cases are stilbene, as well as azobenzene and their derivatives. The reversible transformation between the *trans*– and *cis*–configuration could be modulated by light or heat.

With research and development for more than a century, azobenzene has the most extensive applications in dyes and pigments [15]. Because of their efficient *trans*–*cis* isomerization with appropriate light irradiation, large different molecular geometry, and dipole moment of *trans*– and *cis*–isomers, azobenzene and its derivatives are excellent candidates for molecular photoswitches. Benefitting from easy synthesis [4], fast photoisomerization, relatively high photostationary state (PSS) and quantum yields, and low rate of photobleaching, azobenzene compounds are widely applied in photochemical molecular switches [16], biology [17,18,19,20], solar thermal fuels [21], photopharmacology [22,23], polymers [24,25], soft actuators [26], and other fields.

Because of the small difference in electron delocalization between the *trans* and *cis* isomers, the changes in colors and absorption spectra between the two isomers are typically rather small. The general color of azobenzene is yellow, and different substitutions or linkers yield a bathochromic shift, resulting in a display of orange or red colors [27,28,29]. The maximum absorption wavelength of the *cis*–isomer shows a hypsochromic shift compared to the *trans*–isomer upon isomerization reaction of azobenzene derivatives, which are considered to be the nature of negative photochromism [30,31,32]. This property can broaden their application in biology and medical fields as in vivo photoswitches [20,33,34,35,36].

**Scheme 5:**
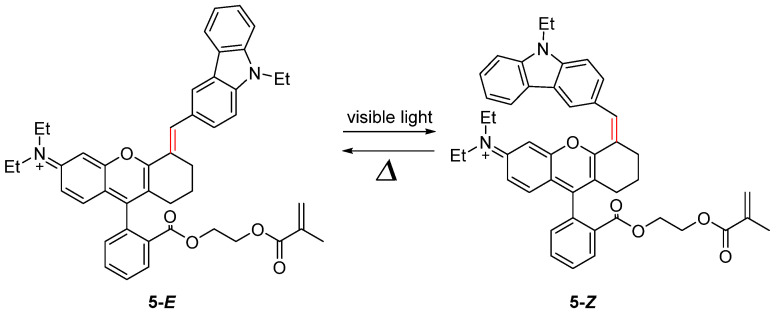
Photoisomerization reaction of compound **5**.

Recently, compound **5** (Scheme 5), which is based on stilbene and incorporates a blue cyanine dye, has been observed to transition from the blue–colored *E*–form (λ_max_ = 650 nm) to light blue–colored *Z*–form (λ_max_ = 420 nm) [37]. This change can be attributed to the co–planar *E*–form and the twisted *Z*–form. The absorption tail of the *E*–form extends into the NIR range (λ > 750 nm). Photochemical quantum yields, *Φ_E-Z_* and *Φ_Z-E_*, are 0.046 and 0.015 in toluene, respectively. Due to visible light excitation and significant differences in molecular geometry and dipole moments between the *trans*– and *cis*–isomers, compound **5** can be considered a promising molecule for applications in biological and medical fields to induce high activity modulation.

### 2.3. Salicylideneaniline (Anils)

Salicylideneaniline derivatives, also called anils, are probably the most studied photochromic compounds with a proton transfer process [38,39]. With thorough study starting from 1964, the significant characterizations, including the proton transfer process, photochromic properties, and photochromism in different media, have all been thoroughly investigated [40,41,42]. Generally, UV light irradiation induces an intramolecular proton transfer process. The typical tautomerism from the enol form to the *cis*–keto form in the excited state can be referred to as excited state intramolecular proton transfer (ESIPT). The process described above can also be considered as phototautomerism, proceeding within a few picoseconds in solution, and a few hundred picoseconds even in solid state [42,43,44,45].

**Scheme 6:**
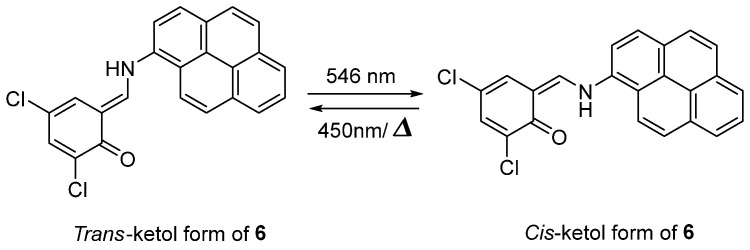
Phototautomerism of compound **6**.

Based on the skeleton of anils, *N*–(3,5–dichlorosalicylidene)–1–aminopyrene **6**, which exhibits photo–reversible negative photochromism in a pure solid powder state, was investigated (Scheme 6) [46]. The color change, as well as a blue shift in the UV–vis absorption spectra, was observed visually upon visible light irradiation (λ = 546 nm), along with a photo–tautomerism from the *trans*–keto form (deep orange) to the *cis*–keto form (slight yellow) interconversion. Furthermore, the stability of the *trans*–keto form under ambient condition broadened the application of this material in information storage, photoswitches, displays, and sensors.

### 2.4. Dihydropyrenes

**Scheme 7:**
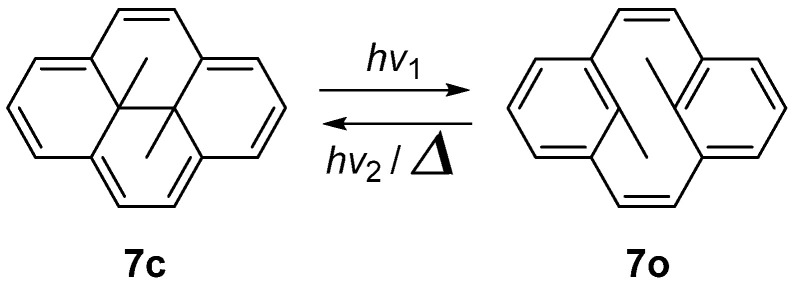
Photoisomerization of dihydropyrene **7**.

Similar to a stilbene linked with an ethylene group, dimethyldihydropyrene exhibits negative photochromism, transitioning from a colored closed isomer (**7c**) to colorless open isomer (**7o**) (Scheme 7). The relative stability of the open and closed isomers is inverted from DAE, because of the stabilization at aromatic 14π–electron system in the closed form **7c** [47].

**Scheme 8:**
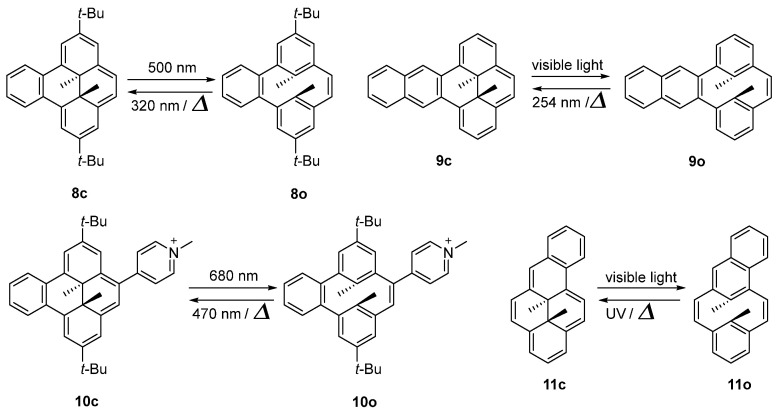
Molecular structures and photochromic reactions of **8**–**11**.

Benzannelation in the [e] position of pyrene hampers the thermal back reaction from open isomer to closed isomer (Scheme 8). Dimethyldihydrobenzo[*e*]pyrene (**8c**) undergoes a facile photoisomerization reaction from the stable purple closed isomer to the open isomer (**8o**) under visible light irradiation (λ > 400 nm) with a ring opening quantum yield (*Φ_c-o_*) of 4.2 × 10^−2^. Notably, the crystalline deep red/purple **8c** would also completely convert to the colorless open isomer **8o** upon visible light irradiation. Meanwhile, the **8o** could completely revert to closed isomer **8c** with UV light irradiation (λ < 350 nm) or slow thermal back reaction [48]. Furthermore, the thermal back reaction rate from naphtho[e]dihydropyrenes (**9o**) to the closed isomer (**9c**) was even sluggish [49].

Moreover, a benzo[*e*]–fused dimethyldihydropyrene compound (**10**) bearing a methyl–pyridinium electroacceptor group was designed [50]. Both the photo–opening and the reverse photo–closing processes can be triggered by visible light illumination and proceed with high quantum yields (respectively, 14.5% yield at λ = 680 nm and quantitative quantum yield at λ = 470 nm, in water). Remarkably, depending on the nature of its counter anion, this system is able to operate in organic and aqueous solvents, with a high fatigue resistance under aerobic conditions.

However, fusion at the [a] position of pyrene gives completely different results. The thermal back reaction from **11o** to **11c** is much faster than normal dihydropyrenes. The formation of **11o** can only be successfully observed through laser flash studies, but rapidly reverted to **11c** [49]. The results described above are consistent with the activation energy barrier between **11o** and **11c** evaluated by DFT calculations [49,51,52].

**Scheme 9:**
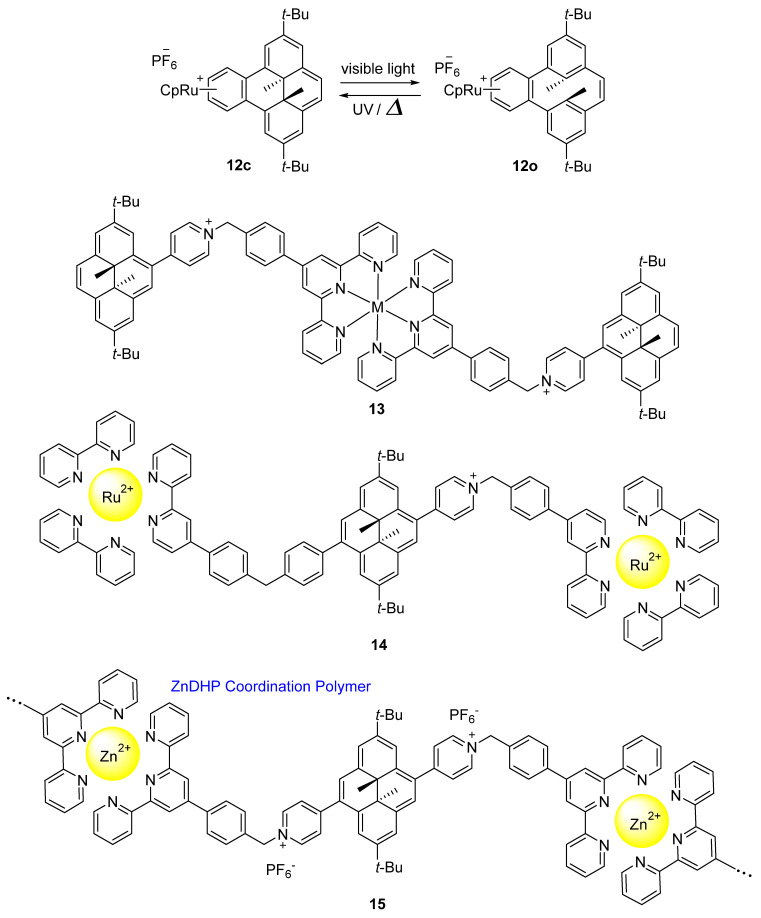
Molecular structures of dihydropyrene metal complexes **12**–**15**.

The investigation of the negative photochromic properties of dihydropyrene compounds was also achieved by the complexation of dihydropyrenes with metal ion (Scheme 9). The purple–colored complex **12c** was obtained according to the complexation reaction of **8c** with ruthenium (II) cyclopentadienyl. The visible light–induced ring–opening reaction of **12c** to form **12o** is slower compared to the precursor **8c** along with a modified thermal ring closure reaction rate. However, there is no obvious effect of the complexation on the ring closure reaction upon UV light irradiation [53].

Dimethyldihydropyrene–appended metal complex **13** exhibits more efficient negative photochromism when the ligand moiety and the photochromic unit are connected through a pyridinium bridge (*Φ* = 0.06) [54]. Isomerization of **14** occurs between its closed and open isomers upon irradiation with red (λ = 690 nm, *Φ* = 0.08) and blue light (λ = 460 nm, *Φ* = 0.21), respectively (Scheme 9) [55]. A stimuli–responsive coordination polymer ZnDHP (**15**) particles exhibits a photochromic behavior based on the reversible conversion of “closed” dihydropyrene units into “open” cyclophanediene. Ring opening in the condensed phase was triggered by visible light (λ > 450 nm) illumination, while back–conversion was performed by either heating or irradiation with UV light. The absorption tag of this ZnDHP coordination polymer can be extended to the NIR region, which forces this compound to undergo NIR light–responsive photochromism [56].

**Scheme 10:**
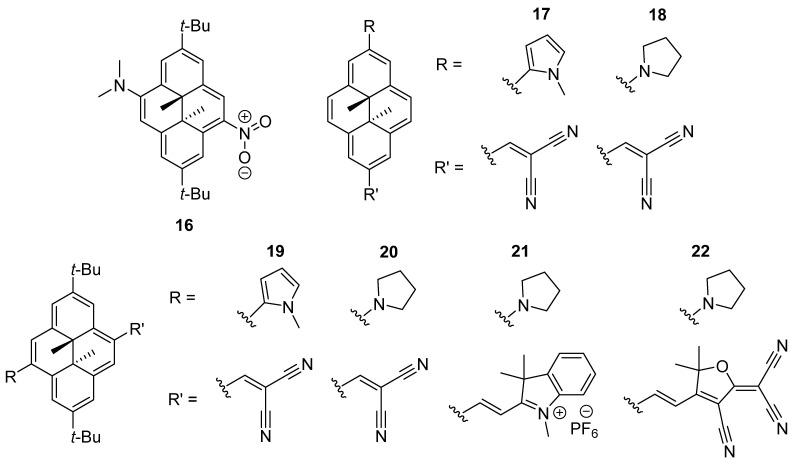
Donor–acceptor functionalization of dihydropyrene compounds **16**–**22**.

NIR light–responsive dihydropyrene photoswitches were also reported by Hecht and coworkers. A direct, one–photon NIR photoswitch based on a dihydropyrene skeleton was designed and investigated. The fine thermal half–lives at body temperature and NIR light–responsive isomerization of the electron donor–acceptor *tert*–butyl–dihydropyrene derivative **16** can be modulated by altering the polarity of the solvents (Scheme 10) [57]. The quantum yields for the photoinduced ring–opening of **16** were measured at 579 nm and 25 °C. In nonpolar solvents, the quantum yields are unexpectedly high (*Φ* = 0.28 *n*–heptane and *Φ* = 0.18 in toluene), while in rather polar solvents, such as DCM, acetonitrile, and DMSO, the quantum yields are somewhat lower (*Φ* ≈ 0.06) yet still high considering dihydropyrenes.

Moreover, two series of direct one–photon NIR light–responsive dihydropyrene compounds (**17**–**22**) with strong donor–acceptor substituents at *pseudo*–*para*–positions of the parent molecule were also established (Scheme 10). Their negative photoisomerization reactions can be initiated completely by far–red or NIR light, leading to the formation of the metastable cyclophanediene isomers [58]. Upon NIR light irradiation for the photochromic compounds described above, the maximization of both NIR one–photon absorption cross–section and photoisomerization efficiency can be achieved along with a reasonable thermal stability of the metastable ring–opening isomer, i.e., cyclophanediene isomer. Generally, the donor–acceptor substitution of dihydropyrenes enhances the quantum yield of the ring opening proceeding via the first excited state. The experimental investigations open up a new avenue for near–infrared light–responsive dihydropyrene photoswitches, applicable in biological and medical functions. Dihydropyrene photoswitches have also been applied for photoresponsive hydrogen–bonded organic frameworks [59].

### 2.5. Donor–Acceptor Stenhouse Adducts (DASAs)

As typical negatively photochromic compounds, donor–acceptor Stenhouse adducts [7] were discovered by Read de Alaniz and coworkers in 2014 following the foregoing research of Stenhouse salts [60,61].

The representative molecular structures of DASAs are those in which an electron donor group connects with an electron acceptor group (either Meldrum’s acid or 1,3–dimethyl barbituric acid) through a triene (“polymethine”) bridge. This molecular structural characteristic introduces this kind of compound as a push–pull system. Thus, they have a strong absorption band in the visible region due to the π–π* transition. In contrast, the photoinduced formation of the cyclopentenone isomer loses the π–π* transition, and its absorption band is located in the UV region. Along with the photochromic reaction, the color changes from colored π–extended isomer to colorless compact isomer. Thus, this behavior causes the DASAs to undergo negative photochromism.

**Scheme 11:**
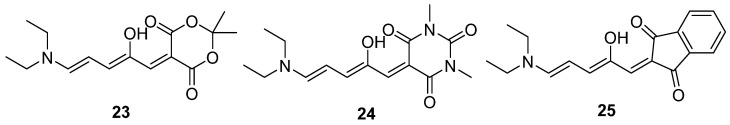
Structures of first–generation DASAs **23**–**25**.

The maximum absorption wavelengths of open isomer DASAs **23**, **24**, and **25** are observed at 545 nm, 570 nm, and 600 nm (Scheme 11). Their corresponding water–soluble photoinduced cyclopentenone isomers were zwitterionic with a unique absorption located in the UV region. The quantum yields of **23** and **24** in toluene are 0.21 and 0.106, respectively [62]. However, the first–generation DASAs face limitations in applications due to restricted wavelength tunability, solvent–dependent reversibility of photoisomerization, and lack of photoreactivity in solid–supported matrices.

**Scheme 12:**
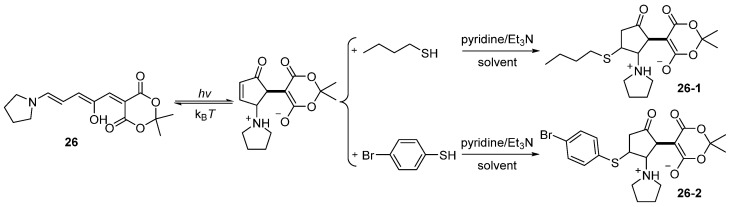
Selective reaction of DASA **26** with thiol–Michael addition.

Furthermore, the cyclopentenone isomer of the first–generation DASA **26** was successfully trapped with 1–butanethiol and *p*–bromothiophenol, leading to the formation of **26-1** and **26-2**, respectively (Scheme 12). This ligation reaction demonstrated that the photochemical properties can be modulated by the selective chemical reaction [63].

In 2016, the second–generation DASAs were designed to address the limitations of the first–generation DASAs [64]. The primary distinction between them lies in changing the donor moiety from dialkylamine to aniline derivatives.

**Scheme 13:**
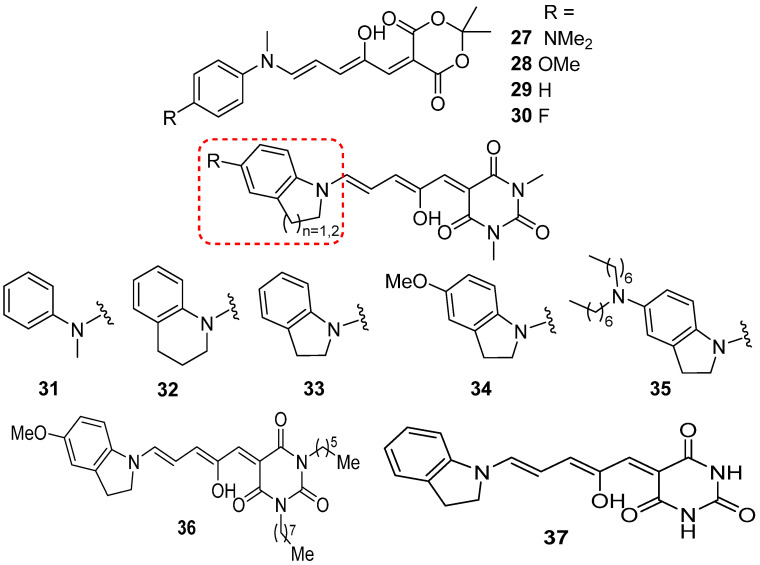
Structures of second–generation DASAs **27**–**37** (Red frame indicates the substituents below which corresponding to compounds **31**–**35**).

The absorption bands for 4–functionalized *N*–methylanilines are located at different wavelengths, ranging from 573 nm to 564 nm and 560 nm and 556 nm, depending on the electron–donating or accepting properties of substituents from NMe_2_ (**27**) to OMe (**28**), H (**29**), and F (**30**) (Scheme 13) [64]. Furthermore, the maximum absorption wavelength of DASAs with *N*–methylaniline (**31**), tetrahydroquinoline (**32**), indoline (**33**), *p*–methpxy–1–indoline (**34**), and *N*,*N*–dialkyl group at the *para*–position of indoline (**35**) are located at 582 nm, 599 nm, 615 nm, 629 nm, and 669 nm, respectively. The bathochromic shift in absorption spectra of compounds **34** and **35**, compared to the parent compound **31**, is likely due to the enhanced hybridization of molecular orbitals between the electron donor and acceptor groups (Scheme 13) [65]. The quantum yield of 34 in toluene is 0.145 [62].

DFT calculations clearly highlighted the significant difference in maximum absorption between these two generations of DASAs. The dihedral angle of cyclic indoline (**33**, dp_D-A_ ≈ 0°) is much smaller compared to acyclic derivatives (**31**, dp_D-A_ ≈ 40°). This implies higher extension of π–conjugation system. A bathochromic shift of ~30 nm in the absorption band is attributed to a narrower energy gap (*E*_g_) from a more delocalized HOMO and extended π–conjugation. Moreover, the photoisomerization of second–generation DASAs is notably capable in polar solvents as well as in solid polymer matrices.

It is worth mentioning that the wavelength–selective photoswitching of two derivatives was demonstrated by means of the large difference in absorption wavelength between DASAs **29** and **36** [66]. Because of almost no overlap in absorption spectra, **29** with a *N*–methylaniline donor and Meldrum’s acid acceptor group (λ_max_ = 560 nm in toluene) and compound **36** with a *p*–methoxy indoline donor and barbituric acid acceptor group (λ_max_ = 623 nm in toluene) were chosen for wavelength–selective photoswitching. The photoisomerization reaction of compound **36** occurred selectively upon 650 nm light irradiation to change the solution color from violet to pink. In contrast, the light with wavelength at 514 nm induced the photochemical reaction of compound **29** with a color change from violet to turquoise. Importantly, this wavelength–selective photoswitching could also happen in the solid matrix of poly(methyl methacrylate) (PMMA). Based on the molecular structure of second–generation of DASAs, the barbiturate acceptor group of DASA photoswitch (**37**) is active in neuronal (GABA)–type A receptors and alters the firing rate of untransfected neurons in a physiological medium at neutral pH, and can be reversibly photoswitched in acidic aqueous cyclodextrin solutions compatible with drug formulations [67].

**Scheme 14:**
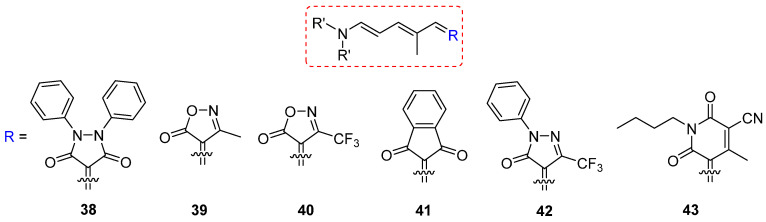
Chemical structures of third–generation DASAs **38**–**43**.

Six DASAs (**38**–**43**) with carbon acid acceptors are termed as third–generation DASAs (Scheme 14) [65]. The absorption of the aforementioned DASAs **39**–**43** was clearly modulated by the acceptor groups, resulting in peak absorptions ranging from 570 nm to 680 nm. Moreover, compounds **40** and **41** were studied for the rewritable paper [68]. The quantum yields of compounds **39** and **41** are 0.4 and 0.1, respectively [69]. The absorption tail of compound **43** can be extended to 735 nm, enabling the photoactivation with 700 nm light and NIR light. This research broadens the applications of DASAs in the biomedical and material fields by utilizing long–wavelength light.

**Scheme 15:**
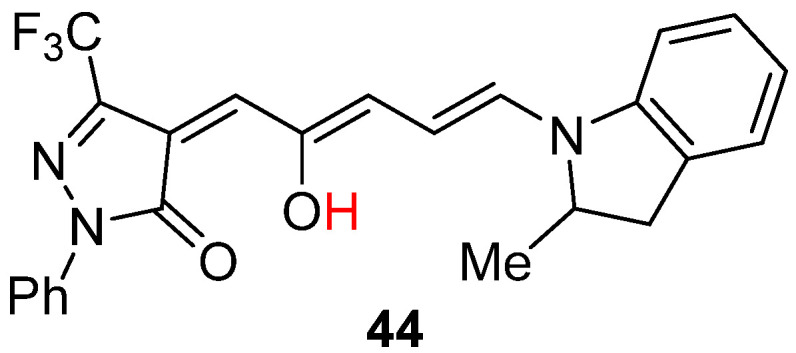
Chemical structures of DASA **44**.

Interestingly, photoisomerization reactivity of a third–generation DASA **44** is highly dependent on the concentration (Scheme 15) [70]. At low concentration, **44** switches efficiently under light irradiation in both liquid solution and in polymer hosts. However, it is dramatically suppressed by increasing concentration. From a two–state kinetic analysis and photophysical measurements, concentration dependence of photoisomerization reactivity is ascribed to the slower electrocyclization than the *cis*–*trans* isomerization of the tri–ethene bridge. The quantum yields of **44** from open isomer to closed isomer were gradually increased along with decreasing the concentration both in solution (toluene and chloroform) and PMMA. This research provides guidance for expanding DASAs’ application at high concentrations.

Capability for the second harmonic generation, SHG, of open forms and the effect of the donor and acceptor groups on the third generations of DASAs (**39**, **41**) have been accessed by means of hyper–Rayleigh scattering measurements. Compounds based on isoxazolone and indanedione display fast thermal back reaction, while those incorporating Meldrum’s acid display longer lifetimes in the closed form. Significant SHG capability was obtained for open form derivatives with appropriate acceptor unit. These third–generation DASAs are expected to display large variation in their nonlinear optical properties upon photoisomerization [69]. Fatigue resistance measurements were also carried out by using different generations of DASAs in solution and polymeric matrices. But different compounds showed different mechanisms, including photo–dependent fatigue mechanism and interactions with additives [71]. Due to the unique properties of DASAs described above, these compounds have potential applications in chemical and thermal sensors [72], drug delivery [73,74], liquid crystals, rewritable paper [68], polymer science [75], and so on.

### 2.6. Imidazole Dimers

The C–N bond of hexaarylbiimidazole (HABI), situated between its two imidazole rings, undergoes homolytic cleavage with light, heat, or pressure. This results in the formation of two triphenylimidazolyl radicals, which spontaneously revert to the initial HABI state. After years of studying HABI compounds, Abe and coworkers discovered the first imidazole dimers that exhibited negative photochromism.

**Scheme 16:**
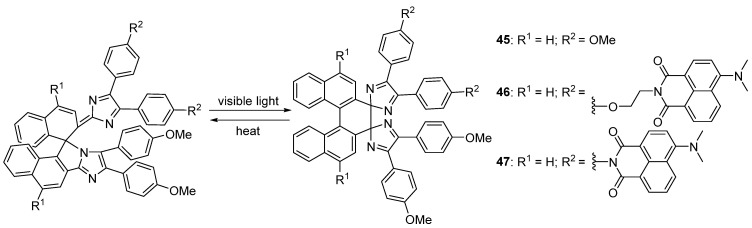
Chemical structures and photoisomerization of imidazole dimers **45**–**47**.

The C–N bond between the nitrogen atom N(1) of the imidazole ring and the carbon atom C(8) at the 1–position of the 1,1′–binaphthyl moiety induced the formation of the pale orange compound **45** with a five–membered ring in the molecular center (Scheme 16). Upon visible light irradiation, the C–N bond of the colored isomer was cleaved homolytically to generate an intermediate imidazolyl radical pair with a half–life of 9.4 μs at 298 K, followed by the formation of a colorless isomer with a new C–C bond between the imidazole rings with radical coupling under the exclusion of light. Certainly, the thermal back reaction recovers the colored isomer in about 20 min along with the original absorption spectrum [76]. The quantum yield of **45** was determined to be 0.03 in hexane.

**Scheme 17:**
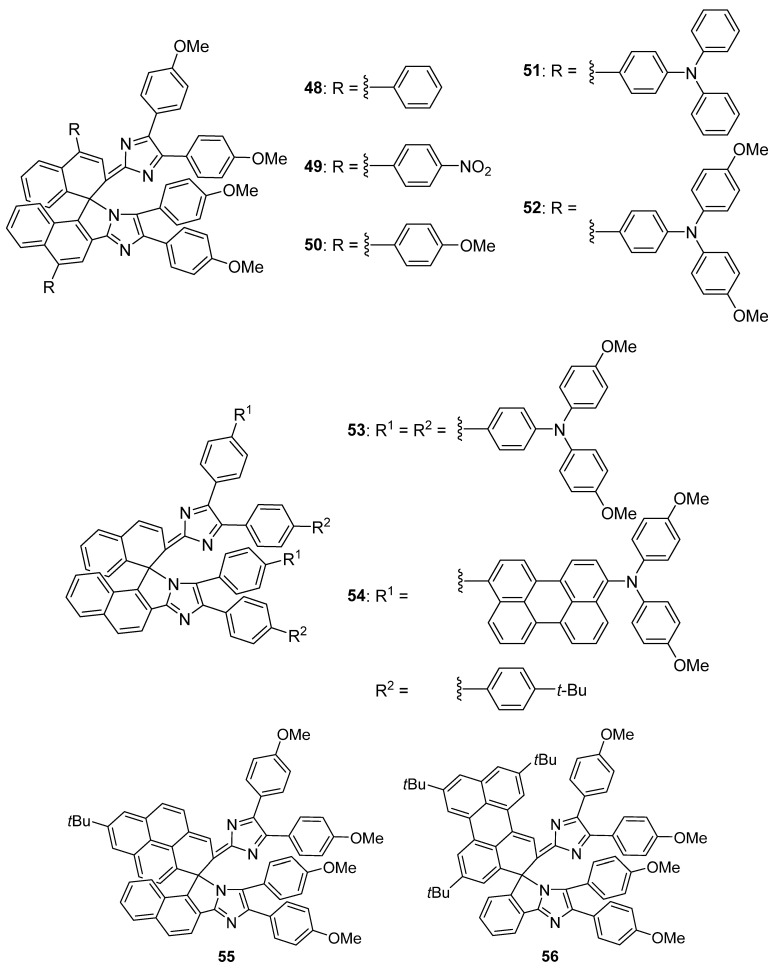
Red or NIR light–responsive negatively photochromic compounds **48**–**56**.

Then, negative photochromic compound **45** was handled as a parent molecule for designing new turn–on mode fluorescence photoswitches **46** and **47** (Scheme 16) [77], as well as red to NIR light–responsive negatively photochromic compounds **48**–**54** (Scheme 17) [78]. Fluorescence quantum yields of **46** and **47** (in benzene, at 298 K) are estimated to be 0.01. Furthermore, 1–(1–naphthyl)pyrenyl–bridged imidazole dimer **55** (Scheme 17) exhibits three–state negative photochromism with a single photochromic unit [79] and 3–phenylperylenyl–bridged imidazole dimer **56** (Scheme 17) demonstrates quantitative and selective bidirectional negative photoisomerization with visible and NIR light irradiation [80].

As a novel photochromic molecule, phenoxyl–imidazolyl radical complex (PIC), which can be recognized as an unsymmetrical HABI, has also been reported by Abe and coworkers [81]. This photochromic molecule consists of three parts: an aromatic linker, a disubstituted imidazole moiety, and a 4H–cyclohexadienone ring. Upon light irradiation, the homolytic cleavage of the C–N bond between the imidazole ring and 4H–cyclohexadienone ring forced the formation of the open isomer with a phenoxyl radical and an imidazolyl radical. As a classic T–type photochromic compound, the colorless open form thermally recovers to the initial colored closed form with a half–life ranging from tens of nanoseconds to seconds.

**Scheme 18:**
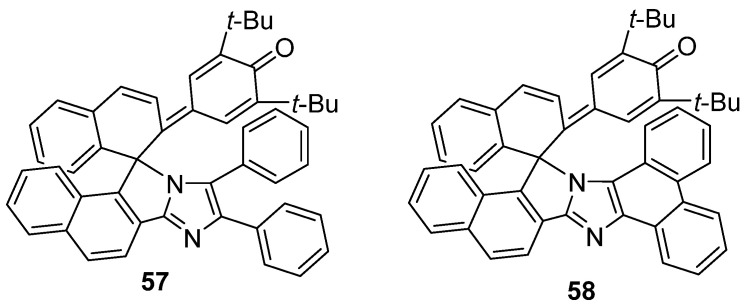
Chemical structures of PICs **57** and **58**.

With the replacement of one imidazole ring of HABI by a corresponding phenol moiety, 1,1′–binaphthyl–bridged phenoxyl–imidazolyl radical complexes (PICs) **57** and **58** were designed (Scheme 18). The yellow–colored compounds **57** and **58** have similar UV–vis absorption spectra with a broad absorption band from the UV region to 500 nm. Both compounds exhibit negative photochromism with remarkable color changes upon visible light irradiation [81]. By using 1,2–bis(2–methylbenzo[*b*]thiophene–3–yl)–perfluorocyclopentene as a standard, the quantum yields of **57** and **58** were determined to be 0.09 and 0.08, respectively.

**Scheme 19:**
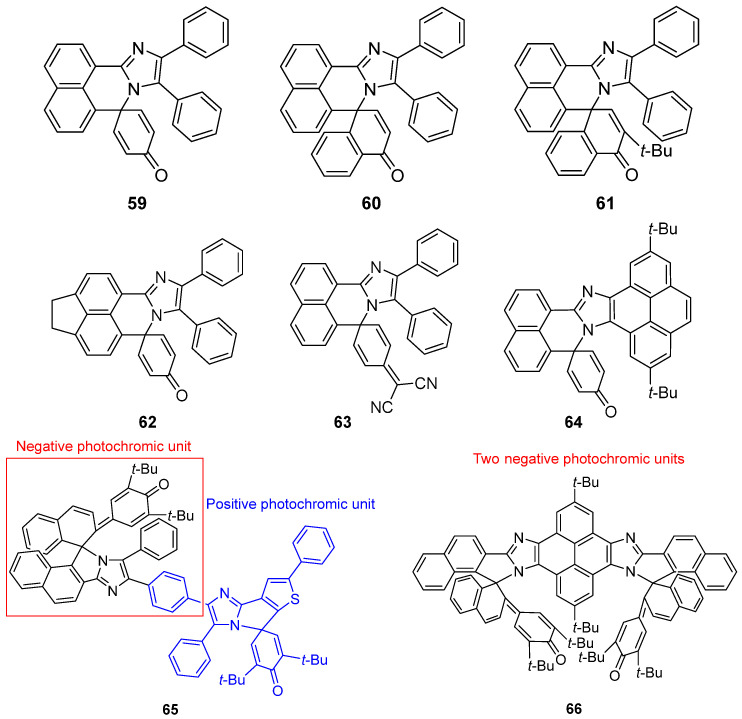
Molecular structures of compounds **59**–**66**.

The characteristic absorption bands of naphthalene–bridged PICs **59**–**61** are situated around 360 nm (Scheme 19) [82]. A hypochromatic shift was clearly observed upon irradiation with 365 nm light in Me–THF. This character leads to the display of typical negative photochromism in compounds **59**–**61**. The conversion efficiencies of **59**, **60**, and **61** were estimated to be 0.18, 0.06, and 0.04, respectively, by using benzophenone as a standard. From the experimental and theoretical investigations of naphthalene–bridged PICs **62**–**64**, it is evident that extending the π conjugation can significantly enhance the photosensitivity and thermal back reaction rate (Scheme 19) [83].

Furthermore, light intensity–dependent biphotochromic molecules composed of a negative and a positive photochromic unit (**65**) [84] and two fast negative photochromic PIC units (**66**) [85] will further expand the development and application of negative photochromic molecules (Scheme 19).

### 2.7. Spiropyran and Spirooxazine

**Scheme 20:**
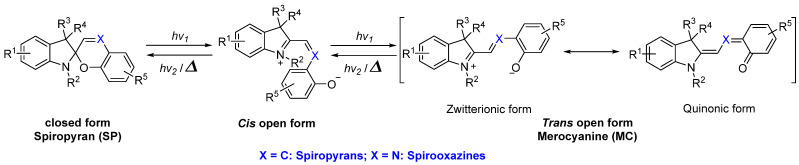
Chemical structures and photochromism of spiropyrans and spirooxazines.

Spiropyrans and spirooxazines are typical T–type photochromic compounds [3]. According to the structural properties of closed and open form isomers, the precondition of them showing negative photochromism is that the open isomer should be more thermal stable compared to the closed isomer (Scheme 20). The most common method for stabilizing the open form is connecting the electron–withdrawing substituents to the benzopyran/phenolate unit in order to delocalize the negative charge throughout the molecule.

The first and most studied spiropyran molecule **67** with nitro groups as electron–withdrawing substituents on a benzopyran moiety was synthesized in 1952 [86] and further studied by crystallographic structural analysis, proton nuclear magnetic resonance, spectroscopic measurement, and so on, in the following decades [87,88,89]. The quantum yield of compound **67** is 0.40. After compound **67**, a number of spiropyrans showing negative photochromism have been designed.

**Scheme 21:**
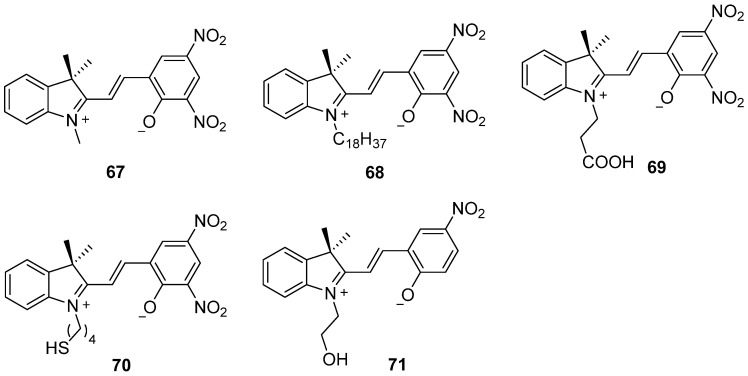
Chemical structures of dinitro–substituted spiropyrans **67**–**71**.

Dinitro–substituted spiropyrans (**67**–**71**) with different pendants connecting with a nitrogen atom at the indoline unit exhibit negative photochromism (Scheme 21). The negative photochromic reaction of *N*–octadecyl–substituted spiropyran **68** occurs in both solution and Langmuir–Blodgett film [90,91]. The first ultrafast photo–dynamics of the ring closure kinetics was investigated by using negative photochromic spiropyran **69**. The quantum yield from the closed to the open form was around 0.44 in acetonitrile. This study reveals the most likely mechanism of the ring closure reaction through a singlet manifold rather than triplet state [92]. With the thiol group binding to the gold surface, the *N*–alkylthio derivative **70** has been used as a reusable sensor for antigen–antibodies due to the reversible photochromic reaction [93]. With a hydroxyl linking group to cotton fabrics through one–bath and coating methods, synchronous photochromic and fluorescent color–changing smart textiles have been developed by using spiropyran **71** [94]. The negative photochromic cotton fabrics can promptly reach color saturation within 12 s. The photochromic cotton fabrics can respond to both visible and UV light, and also have fluorescent color–changing properties. The prepared photochromic fabrics seem to have wide applications in the areas of UV protection, imaging information switching, as well as information encryption and decryption.

**Scheme 22:**
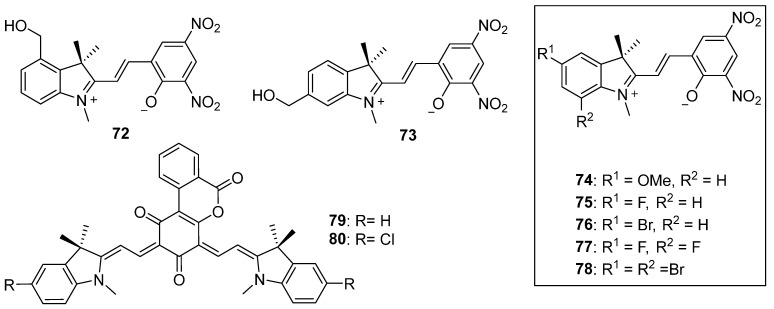
Chemical structures of spiropyrans **72**–**80**.

Spiropyran **67** and its derivatives **72** and **73** [95] with a hydroxymethyl group at either the 4– or 6–position have been employed as practical sensors for selective detection of thiol–containing amino acids with different mechanisms (Scheme 22). Interestingly, the maximum absorption wavelength of the open isomers and the negative photochromic properties of spiropyrans **74**–**78** with different substituents at both the 5– and 7–positions are significantly affected by the substituents and the solvents (Scheme 22). Spiropyran **76** in chloroform and spiropyran **78** in both chloroform and acetone exhibit positive photochromism; they show negative photochromism in DMSO solution [96]. This result indicates the significant influence of the polar medium for stabilizing the open isomer of the spiropyrans. In DMSO solution, both nonsymmetric bis–spiropyran derivatives **79** and **80** are fully open in the merocyanine form with maximum absorption bands at 477–479 nm and 528–562 nm, respectively. The visible light (λ = 546 nm) irradiation then induced the formation of the closed spiro–type isomers along with color–fading behavior [97].

**Scheme 23:**
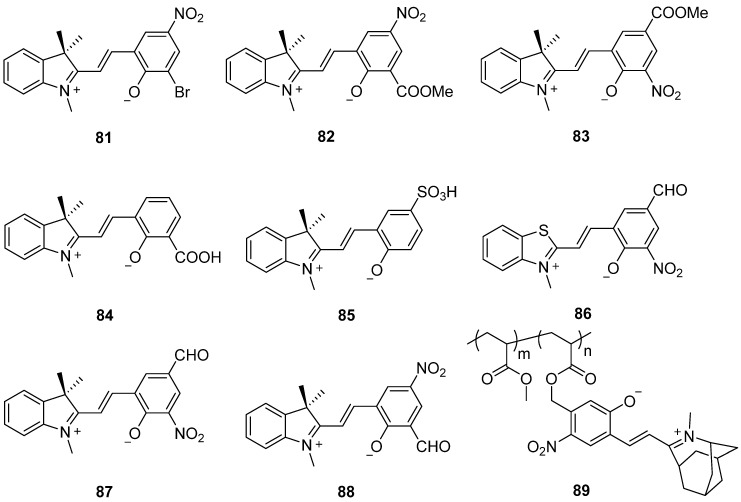
Chemical structures of spiropyrans **81**–**89**.

Featuring a bromine atom instead of a nitro group in the 8′–position, compound **81** demonstrates the presence of 60% of the open isomer in the DMSO–*d6* solution (Scheme 23). This indicates that the open isomer is more stable than the closed isomer in solution at room temperature [98]. The open isomers of **82** and **83** with ester substituents at the 8′– or 6′–position are more stable in polar solvents, such as ethanol and *N*,*N*–dimethylformamide (Scheme 23) [99]. Compound **84** exhibits negative photochromism due to the intramolecular hydrogen bond formation. This bond is formed between the hydrogen atom on the carboxyl group and the oxygen atom on phenolate, which significantly stabilizes the open isomer. As a result, it transitions from a deeply colored isomer to an unstable colorless isomer in polar solvents. The quantum yield of the photoconversion from colored isomer to colorless isomer is 1.7 × 10^−3^ in ethanol [100,101]. The red– or reddish–orange–colored compound **85** with sulfonic acid at the 6′–position exhibits negative photochromism in both polar protic solvents and AOT reversed micelles (Scheme 23) [102].

Similar to compounds **81**–**85**, spiropyrans **86**–**88** of the indoline and benzothiazole series with electron–withdrawing substituents exhibit negative photochromism in solutions. The merocyanine isomers (open forms) are fully characterized by long–wavelength absorption bands with maxima in the area of 542–544 nm in DMSO solution. The photochemical quantum yields of both bleaching and coloration reactions are in the range of 0.013–0.49 and 0.10–0.58, respectively. Furthermore, compounds **87** and **88** exhibit new chromogenic properties associated with the barochromic effect in the gas phase [103]. Visible–light–driven photoswitchable fluorescent polymers by copolymerization of novel negative photochromic spiropyran monomer **89** and methyl acrylate are developed by Cui and coworkers [104]. The obtained materials display high brightness and on–to–off switching ratio, outstanding photoreversibility, and excellent photostability. Furthermore, these materials can be applied in high–precision and antifatigue photo–rewritable fluorescence patterns, advanced anti–counterfeiting seals, and information encryption.

**Scheme 24:**
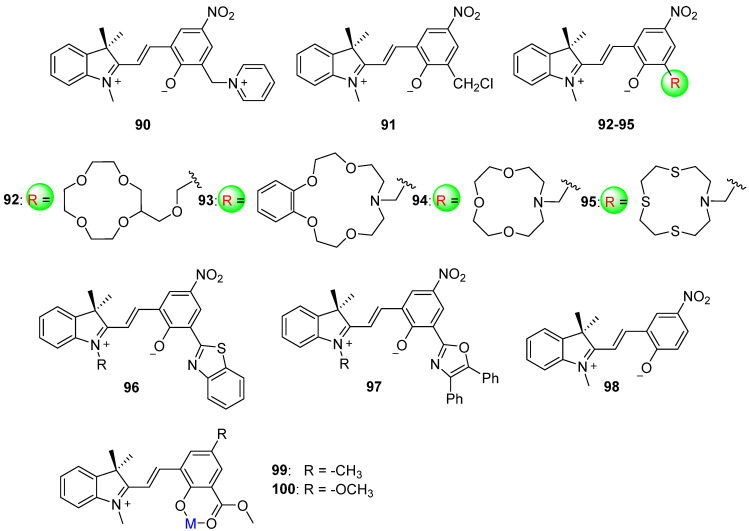
Chemical structures of spiropyrans **90**–**100**.

Various substituents at the 8′–position have been employed as bidentate ligands to bond with metal ions, enhancing the stability of the phenoxide unit in the open isomer and facilitating its conjugation (Scheme 24). Spiropyrans **90** [105] and **91** [106] with pyridine and chloromethyl groups at the 8′–position exhibit negative photochromism due to their chelating functions. The crown–structured spiropyrans **92**–**95** demonstrate metal ion–responsive photochromism. A strong interaction between the crown ether moiety and metal ion results in negative photochromism, while a weaker interaction affords positive photochromism [107]. The azole unit at the 8′–position stabilizes the open isomer of spiropyrans **96** and **97** in the presence of a metal ion, resulting in negative photochromism [108,109]. The quantum yields of photobleaching reaction for derivatives of **96** are 0.21, 0.27, and 0.53 in acetonitrile solution with a methyl group, propyl group, and tertiary butyl as R groups, respectively. The monosubstituted compound **98** with one nitro group at the 6′–position exhibits normal positive photochromism. However, when malonic acid is added to the acetone solution, the protonated compound **98** exhibits negative photochromism [110]. The quantum yield of photoconversion of **98** from colored open form to colorless closed form is as high as 0.73 in acetone solution. Additionally, the interaction between compound **98** and mesoporous silicas is able to accelerate the negative photochromism [111,112]. Compounds **99** and **100** with electron–donating groups and coordinative methyl ester show remarkable color change after the addition of metal ions. The dynamic coordination bonds stabilize the open forms of compounds **99** and **100** and the maximum absorption wavelengths for **99** and **100** are 527 nm and 561 nm, respectively. Hence, the metal complexes of spiropyrans **99** and **100** exhibit negative photochromic properties and are responsive to visible or even daily sunlight [113].

Besides that, heterofused spiropyrans, spirooxazines, spirothiopyrans, linked spiropyrans, bispiropyrans, and so on, showing negative photochromism, have already been summarized in a minireview paper [10].

### 2.8. Tricyanofuran and Hydroxytricyanopyrrole

**Scheme 25:**
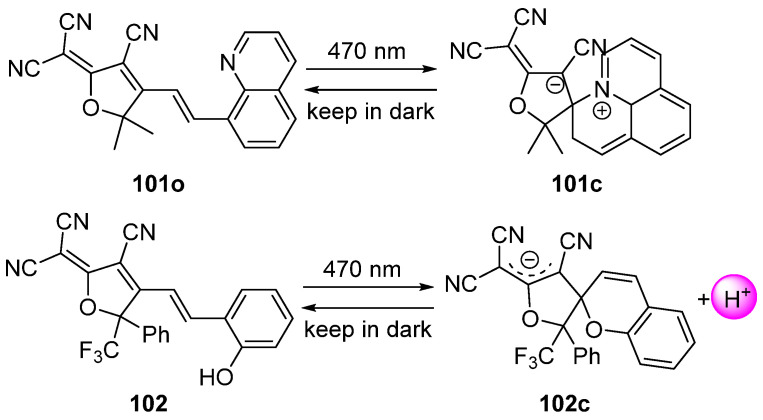
Photochromic reactions of TCF compounds **101** and **102**.

Different negatively photochromic compounds have been designed and studied based on the molecular structure of spiropyran (Scheme 25). When exposed to visible light at 470 nm, the yellow, neutral, and thermostable isomer **101o**, featuring a tricyanofuran (TCF) unit and a quinoline moiety linked by a double bond bridge, transfers to the slight yellow, metastable cyclic zwitterionic isomer **101c** with a high photoinduced transformation efficiency [114]. Afterward, the cyclized zwitterionic isomer **101c**, which has an absorption maximum (λ_max_) at 310 nm, gradually reverts to the open isomer **101o** at room temperature. Based on the decrease in the absorption of **101o** in the first 60 s, the quantum yield was estimated to be 0.197. The TCF–type photoacid generator **102o** transfers from low–acidity open isomer to the metastable high–acidity isomer **102c** upon visible light irradiation (λ = 470 nm). The quantum yield of the photoreaction of **102o** was evaluated to be 0.24 [115]. This class of compounds is expected for use as sulfatase activity–based probe [116], synergistic phototherapy [117], nonlinear optical chromophores [118,119], and so on.

Furthermore, a series of hydroxytricyanopyrrole (HTCP) acceptors based on the TCF structure have been reported by Fedoseev and coworkers. Their key structural deviations of HTCP from TCF are the presence of a hydroxyl group and a NH fragment instead of a methyl group and an oxygen atom, respectively.

**Scheme 26:**
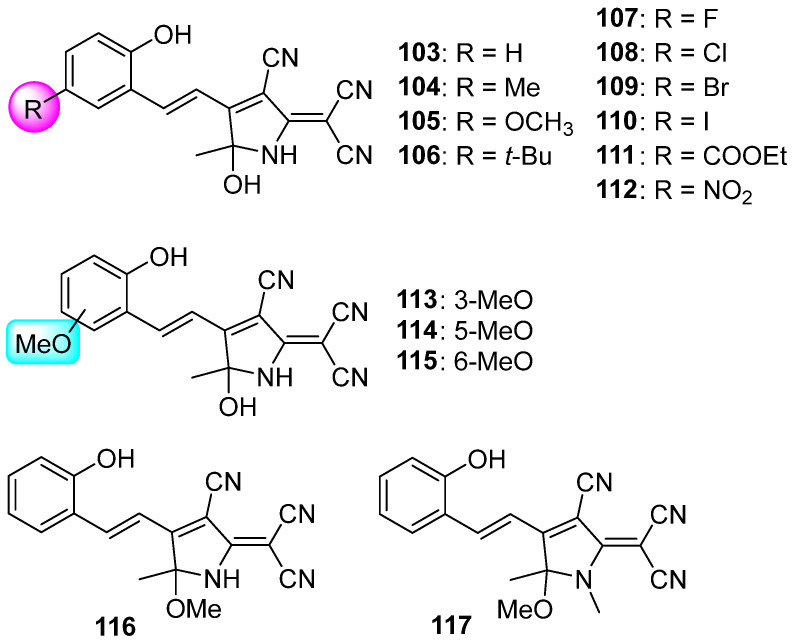
Chemical structures of HTCP derivatives **103**–**117**.

As shown in Scheme 26, the HTCP **103** without any substituents at the benzene ring exhibited an absorption band in the visible region with a maximum at about 426 nm. Visible light irradiation induced a significant decrease in the absorption band in the visible light region and the emergence of a new peak at 315 nm. Indeed, this outstanding characteristic is a hallmark of the negative photochromism phenomenon [120]. Then, the negative photochromic properties of HTCP derivatives (**104**–**115**) with different substituents at the benzene unit have been adequately investigated [121,122]. The excellent structural plasticity indicates a broad prospect for fine–tuning of the photochromic properties of HTCP derivatives. Various approaches for the directed modification of the pyrrole ring of the HTCP photochromes were established by M. Y. Belikov and coworkers [123]. Compounds **116** and **117** with directly functionalized first and fifth positions of the pyrrole ring exhibit negative photochromism along with maximum absorption wavelength at 434 nm and 431 nm in ethanol solution. Furthermore, HTCP derivatives can also be applied for fluorescent donor–acceptor chromophores [124,125,126], fluorescent probes [127], and so on.

### 2.9. Others

Indigoids and their corresponding derivatives [128], hydrazone switches [129], acene derivatives [130], acylhydrazones [131,132], and so on commendably enrich the community of negatively photochromic compounds.

## 3. Conclusions and Perspectives

Negatively photochromic compounds are not as popular as their positive counterparts, but they are more attractive due to their ability to undergo photoisomerization with visible or even NIR light irradiation, along with their high photoreaction efficiency. In this review, we have provided an overview of various types of negatively photochromic compounds based on different molecular skeletons. Their history, design strategies, photochromic properties, and potential applications were also summarized and demonstrated.

The most general design strategy for negatively photochromic compounds is to change the π conjugation length of the molecular structure, along with the photoisomerization reaction. That is, the photo–induced isomerization reaction forces the entire photochromic molecular structure to change from a long π–conjugation length to a relatively short one. Generally, the photochromic reaction of DAE compounds is induced by ultraviolet light irradiation. The molecular structure changes from the open form isomer to the closed form isomer, along with their corresponding absorption bands located in the ultraviolet and visible regions, respectively. However, the different attachment positions between the ethene bridge and the side–aryl chains allow such compounds to exhibit different photochromic behaviors. That is the main strategy for designing DAE compounds displaying negative photochromism. This research result opens up new possibilities for the applications of DAE compounds in all–organic chiral magnetic switches, anti–counterfeiting materials, and photosensitive liquid crystal materials. Based on the similar photoisomerization reaction mechanism to that of DAE, dihydropyrenes also reveal photochromic reaction with light irradiation or heating. Unlike DAE, the negative photochromic behavior of dihydropyrenes is from the stable, closed isomer, dimethyldihydropyrene, to the metastable isomer, metacyclophanediene. Nonetheless, the design principle for dihydropyrenes to exhibit negative photochromism is almost the same as that for DAE compounds, that is, the stable isomer has relatively long π–conjugation length.

Due to the extended isomer of DASAs having a long π conjugation system, the maximum absorption wavelength of the stable colored isomer is located in visible light region. On the contrary, the absorption spectrum of the compact isomer, which is produced by visible light irradiation to the extended isomer, shows a dramatically hypochromic shift. This property makes DASAs typical negatively photochromic compounds. That is almost the same method and strategy for designing spiropyrans and their derivatives to show negative photochromism. Due to their large molecular structural changes upon visible light irradiation, DASAs and spiropyrans hold great promise for applications in drug delivery, liquid crystal, wavelength–selective photoswitching, and chemosensing. For azobenzene and salicylideneaniline, the pendant aryl and heteroaryl moieties attached to the double bond enable them to exhibit negative photochromism along with isomerization reactions. The visible light–induced isomerization broadens the prospects for in vivo applications. Benefitting from the typical carbon and nitrogen linkage sites between the imidazole unit and aromatic linker of PIC and HABI, these classic T–type photochromic compounds reveal a negative photochromism phenomenon. This unusual negative photochromism may open new avenues in the development of advanced photoresponsive materials.

It is well–known that the future research hotspot is to develop low–energy light–responsive photochromic compounds with high photochromic reaction efficiency. The emergence of negatively photochromic compounds opens up a new research direction for designing such photochromic materials and enhances the application prospect of photochromic compounds to a new level.

## Data Availability

Not applicable.
